# Weakly Supervised Skull Stripping of Magnetic Resonance Imaging of Brain Tumor Patients

**DOI:** 10.3389/fnimg.2022.832512

**Published:** 2022-04-25

**Authors:** Sara Ranjbar, Kyle W. Singleton, Lee Curtin, Cassandra R. Rickertsen, Lisa E. Paulson, Leland S. Hu, Joseph Ross Mitchell, Kristin R. Swanson

**Affiliations:** ^1^Mathematical NeuroOncology Lab, Department of Neurosurgery, Mayo Clinic, Phoenix, AZ, United States; ^2^Department of Diagnostic Imaging and Interventional Radiology, Mayo Clinic, Phoenix, AZ, United States; ^3^Department of Medicine, Faculty of Medicine & Dentistry and the Alberta Machine Intelligence Institute, University of Alberta, Edmonton, AB, Canada; ^4^Provincial Clinical Excellence Portfolio, Alberta Health Services, Edmonton, AB, Canada

**Keywords:** MRI, brain tumors, brain extraction, skull stripping, deep learning, weakly supervised learning

## Abstract

Automatic brain tumor segmentation is particularly challenging on magnetic resonance imaging (MRI) with marked pathologies, such as brain tumors, which usually cause large displacement, abnormal appearance, and deformation of brain tissue. Despite an abundance of previous literature on learning-based methodologies for MRI segmentation, few works have focused on tackling MRI skull stripping of brain tumor patient data. This gap in literature can be associated with the lack of publicly available data (due to concerns about patient identification) and the labor-intensive nature of generating ground truth labels for model training. In this retrospective study, we assessed the performance of Dense-Vnet in skull stripping brain tumor patient MRI trained on our large multi-institutional brain tumor patient dataset. Our data included pretreatment MRI of 668 patients from our in-house institutional review board–approved multi-institutional brain tumor repository. Because of the absence of ground truth, we used imperfect automatically generated training labels using SPM12 software. We trained the network using common MRI sequences in oncology: T1-weighted with gadolinium contrast, T2-weighted fluid-attenuated inversion recovery, or both. We measured model performance against 30 independent brain tumor test cases with available manual brain masks. All images were harmonized for voxel spacing and volumetric dimensions before model training. Model training was performed using the modularly structured deep learning platform NiftyNet that is tailored toward simplifying medical image analysis. Our proposed approach showed the success of a weakly supervised deep learning approach in MRI brain extraction even in the presence of pathology. Our best model achieved an average Dice score, sensitivity, and specificity of, respectively, 94.5, 96.4, and 98.5% on the multi-institutional independent brain tumor test set. To further contextualize our results within existing literature on healthy brain segmentation, we tested the model against healthy subjects from the benchmark LBPA40 dataset. For this dataset, the model achieved an average Dice score, sensitivity, and specificity of 96.2, 96.6, and 99.2%, which are, although comparable to other publications, slightly lower than the performance of models trained on healthy patients. We associate this drop in performance with the use of brain tumor data for model training and its influence on brain appearance.

## Introduction

Magnetic resonance imaging (MRI) has a pivotal role in noninvasive diagnosis and monitoring of many neurological diseases (Fox and Schott, 2004; Bauer et al., 2013). The large amount of data produced in routine patient care has prompted the birth of many studies aiming to automate image analysis tasks relevant to patient care including volumetric analyses (Filipek et al., 1997; Shattuck et al., 2001), tissue classification (Hu et al., 2015, 2017; Kickingereder et al., 2016; Ramkumar et al., 2017), disease staging (Chaddad et al., 2018; Ranjbar et al., 2019b), and localization of pathology (Fox and Schott, 2004; Bauer et al., 2013). To successfully characterize both normal baseline and pathological deviation (Kalavathi and Prasath, 2016) on MRI, non-brain tissues such as fat, skull, eyeballs, eyes, and teeth need to be removed from images, as well as cerebrospinal fluid (CSF) surrounding the brain. As manual annotation of brain tissue in a volumetric MRI is excruciatingly labor intensive, many automatic “whole brain extraction” or “skull stripping” techniques have been introduced in the literature to tackle this need. Separating brain and non-brain tissue has been achieved using edge-based (Somasundaram and Kalaiselvi, 2011; Speier et al., 2011), intensity-based (Ashburner and Friston, 2000; Hahn and Peitgen, 2000), and deformable surface-based methods (Smith, 2002; Jenkinson et al., 2005; Zhuang et al., 2006; Galdames et al., 2012). Atlas-based (Leung et al., 2011) and patch-based (Eskildsen et al., 2012; Roy et al., 2017) methods define the boundaries of the brain by registering images to one or many atlases either on the entire image or on nonlocal image patches. Hybrid methods (Segonne et al., 2001; Rehm et al., 2004) that integrate several of the above approaches have been found (Boesen et al., 2004; Iglesias et al., 2011) superior to any individual method in accuracy at the expense of time efficiency.

However, these methods offer fluctuating accuracies with heterogeneous datasets with varying levels of image resolutions, noise, and artifacts (Kalavathi and Prasath, 2016), and as they are designed for healthy brains, they fail in the presence of pathological conditions on images (Speier et al., 2011). Glioblastoma (GBM), a brain tumor known for its diffuse infiltration, creates serious challenges for most skull stripping methods because of large regions of edema or administration of contrast agents during the examination (Speier et al., 2011). Moreover, GBMs are often cortically localized with abnormalities extending to the edge of the brain and deformities in MRI known as brain shift, which can throw off morphological skull stripping approaches that have rigid assumptions about brain appearance.

Recent success of deep learning has made a lasting impact in computer vision and by extension in biomedical image analysis. Deep convolutional neural networks (CNNs) have shown success in several neuroimaging applications such as MR sequence classification (Ranjbar et al., 2019a), prediction of genetic mutation using MRI (Chang et al., 2018; Yogananda et al., 2019), and tumor segmentation (Işin et al., 2016; Pereira et al., 2016). Naturally, several works have explored the utility of deep learning approaches in MRI skull stripping (Kleesiek et al., 2016; Mohseni Salehi et al., 2017) and have reported high performance on publicly available datasets of normal brains. Given the level of variability that we routinely observe in brain tumor data with respect to image quality as well as shape, size, and the location of abnormalities, rule-based approaches might not be well-suited for skull stripping MRI data in oncology, and there is a need for learning-based approaches for skull stripping MRI of patients with brain tumors. However, labeled training data are scarce in this case as whole-brain labels require substantial time to obtain and have no immediate clinical utility. In the absence of fully ground truth labels, weakly supervised learning, where imperfect and inexact labels are used for model training, offers a more approachable alternative and has previously shown success in segmentation of brain structures on MRI (Bontempi et al., 2020). In this work, we assessed the performance of a weakly supervised three-dimensional (3D) skull stripping approach to generate brain masks for multi-institutional brain tumor data when training data were also brain tumor data. To the best of our knowledge, our work is the first of its kind as no previous study has explored the use of both imperfect labels and pathological MRIs to train a skull stripping model.

The contributions of our work are therefore (1) training a 3D CNN for brain extraction leveraging a diverse set of multi-institutional brain tumor data for model training, (2) use of imperfect automatically generated labels for ground truth, (3) comparison of results across two clinically standard MRI sequences (T1-weighted post injection of gadolinium contrast ([T1Gd] or fluid-attenuated inversion recovery [FLAIR]) used in oncology, and (4) assessing the performance of a skull stripping model trained on brain tumor data on a dataset of healthy subjects.

## Materials and Methods

### Data

#### Brain Tumor Images

Our in-house institutional review board (IRB)–approved repository [described in our previous work; Ranjbar et al., 2019a), which contains more than 70,000 serial structural MR studies of 2,500+ unique brain tumor patients acquired across 20+ institutions, was used as the source of brain tumor data. We included paired pretreatment T1Gd and FLAIR series of 668 adult brain tumor image series. The vast majority of this dataset consists of one imaging time point per patient with available T1Gd and FLAIR series, with the exception of one patient with two time points and another with three, which were also acquired at different institutions. We used patients with paired imaging available to compare model performance across different input combinations without concerns about dataset differences influencing the results. We also excluded post-treatment images from the cohort as brain tumor treatment typically including surgery, radiation, and chemotherapy can have varying effects on the appearance of MRI. Because of the retrospective nature of our database, various anatomical and quantitative MRI sequences were available for our patients, and the availability of a certain sequence was dependent on the decision of the patient's clinical team. We chose to include only T1Gd and FLAIR sequences because of their common use in clinical practice and their prevalence in our database. These series were randomly assigned to 586 training, 52 validation, and 30 test cases. Imaging time points from the same patient were placed in the same data split. As creating ground truth labels for the entire brain on volumetric MRI is very cumbersome and time-consuming, the number of test cases were limited to only 30.

As the data were acquired between 1990 and 2016, many factors varied among samples including field strength and acquisition parameters. We used a number of preprocessing steps to harmonize the data including noise reduction with nonlinear curvature-flow noise reduction (Sethian, 1999), radiofrequency non-uniformity correction reduced using the N4 algorithm (Tustison et al., 2010), resizing to a common matrix size of 240 × 240 × 64 voxels and a voxel resolution of 1 × 1 × 2 mm. The SimpleElastix framework (Marstal et al., 2016) was used to rigidly coregister the FLAIR image to the T1Gd image within each study to enable a comparative experiment of model training on both sequences simultaneously.

#### Brain Tumor Labels

**Figure 1:**
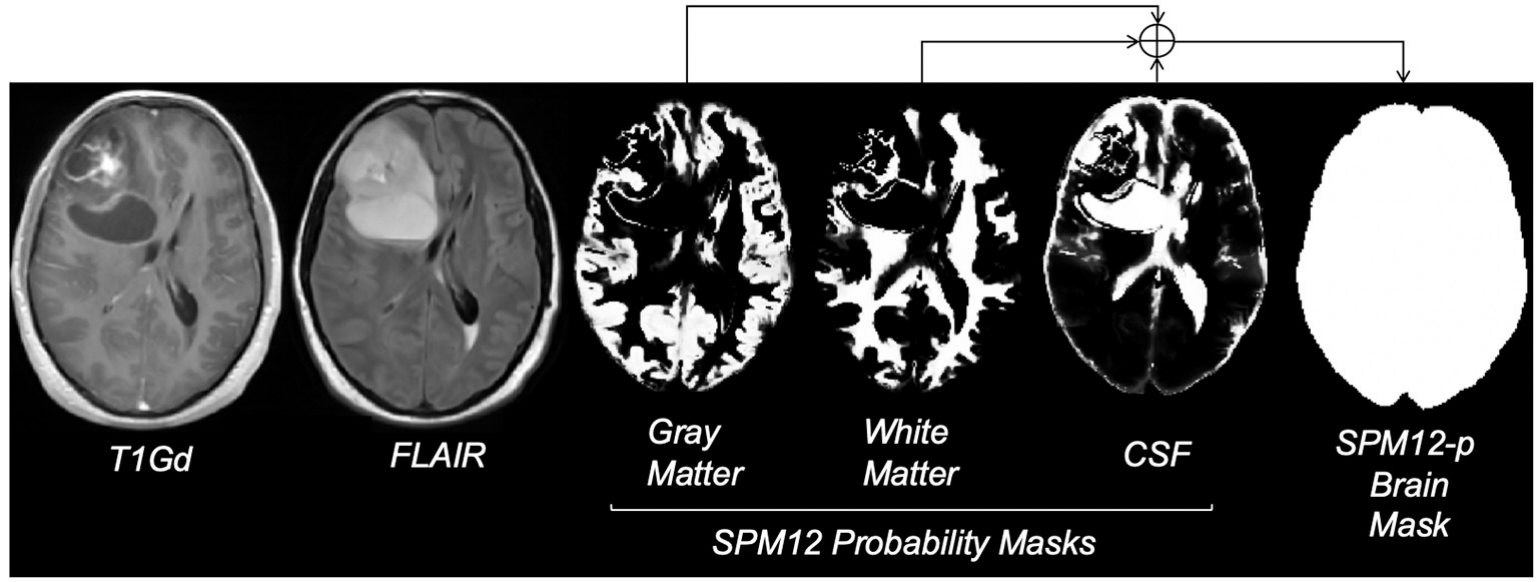
Steps for creating the SPM12-p brain masks; images reflect the MRI of a 29-year-old male brain tumor patient with a diagnosis of GBM. FLAIR refers to fluid-attenuated inversion recovery MRI and T1Gd refers to T1-weighted MRI with gadolinium contrast enhancement. Gray matter, white matter, and CSF probability masks were generated using the SPM12 software. Bright voxels in these masks reflect higher probability. The final brain mask was generated by combining probability masks, using a threshold of 0.7, and minimal post-processing.

Given the large size of our cohort and the time-consuming nature of manual segmentation, we devised an automatic approach to substitute manual delineation of brain masks for model training. We used the Statistical Parameter Mapping (Penny et al., 2011) software SPM12, which contains tools for processing many neuroimaging modalities including structural MRI. SPM12 software generated probability maps for gray matter, white matter, and CSF from all T1Gd MRIs. For each case, the maps were combined into a single map and binarized using 0.7 probability (empirically decided) to generate a brain mask. In some cases, the presence of tumor necrosis resulted in occasional missing areas inside the combined mask, which we accounted for by performing minimal morphological operations erosion followed by dilation to fill in the gaps. The final post-processed result for each brain (referred to as SPM12-p) was stored as a label for model training and validation (Figure 1). SPM12 was run in MATLAB version 2018a, and postprocessing steps were executed in Python 3.6.6. This process was also conducted on test cases to allow for comparison of labels with manual ground truth.

**Figure 2:**
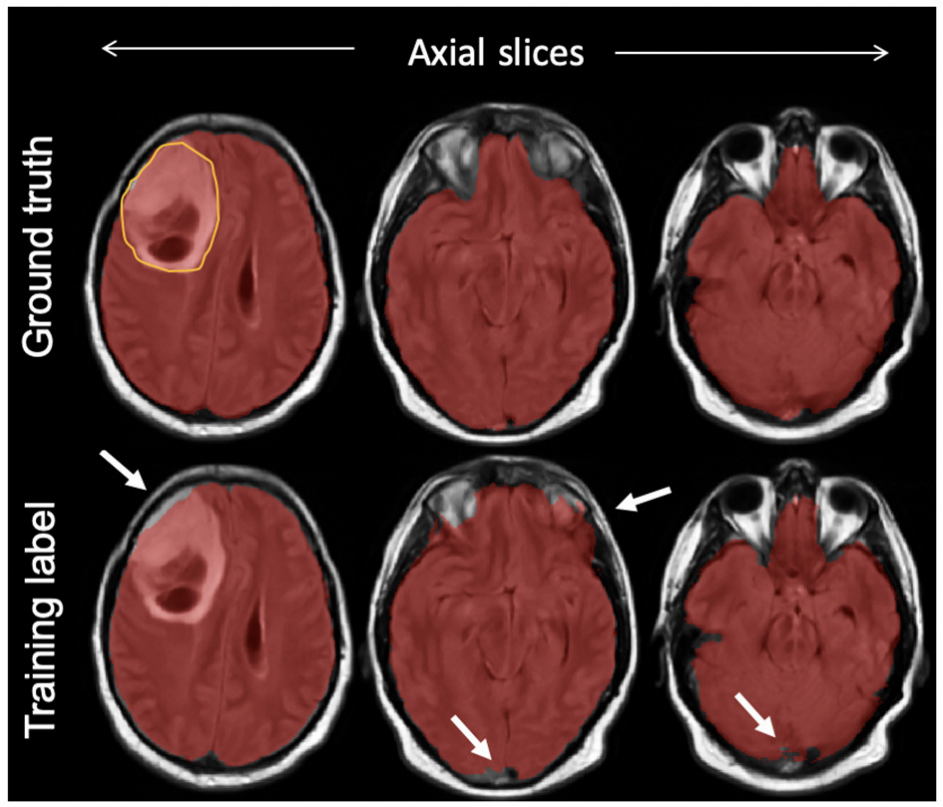
An example of a final training label compared with ground truth; semiautomatically generated training labels were created using SPM12 software. As highlighted with arrows, compared with ground truth delineated manually, the training label included some undersegmentation and oversegmentation particularly around the edges of the brain, but included the bulk of the tumor (outlined on top left slice).

On the test set, we manually segmented brain regions to establish ground truth for estimating model performance. The intracranial volume was defined as the combination of gray matter, white matter, subarachnoid CSF, ventricles (lateral, third, fourth), and cerebellum as suggested by a previous work in the literature (Roy et al., 2017). Manual segmentation was initiated by one of two trained individuals with experience in MRI tumor segmentation using our in-house semiautomatic software. The results were further loaded into the ITK-SNAP (Yushkevich et al., 2006) software version 3.8.0 and corrected manually by a third individual as needed. Figure 2 compares the manual mask and SPM12-p label for one of the test cases.

To further enable comparison with existing atlas-based skull stripping methods in the literature, we generated a third set of labels for the test cases using the Multi-cONtrast brain STRipping method (MONSTR; Roy et al., 2017), a patch-based multiatlas skull stripping method. Although not extensively tested on brain tumor patient data, MONSTR is a benchmark skull stripping approach that was advertised for having success in brain extraction of pathological MRI including patients with traumatic brain injuries and tumors. We refer to these brain masks as MONSTR masks hereon. MONSTR masks were generated using both T1Gd and FLAIR contrasts as inputs.

#### Healthy Subjects Data

The publicly available LONI Probabilistic Brain Atlas Project (LBPA40) (Shattuck et al., 2009) consisting of T1-weighted MRI of 40 healthy subjects was used for evaluation of the model against publicly available benchmarks. The corresponding manually delineated brain masks included in this dataset were used as ground truth. Although training data for this work were entirely brain tumor patients, using this dataset will allow us to contextualize our work within the existing skull stripping literature that have evaluated their approach on MRI of healthy subjects.

### Model Training and Convolutional Neural Network

**Figure 3:**
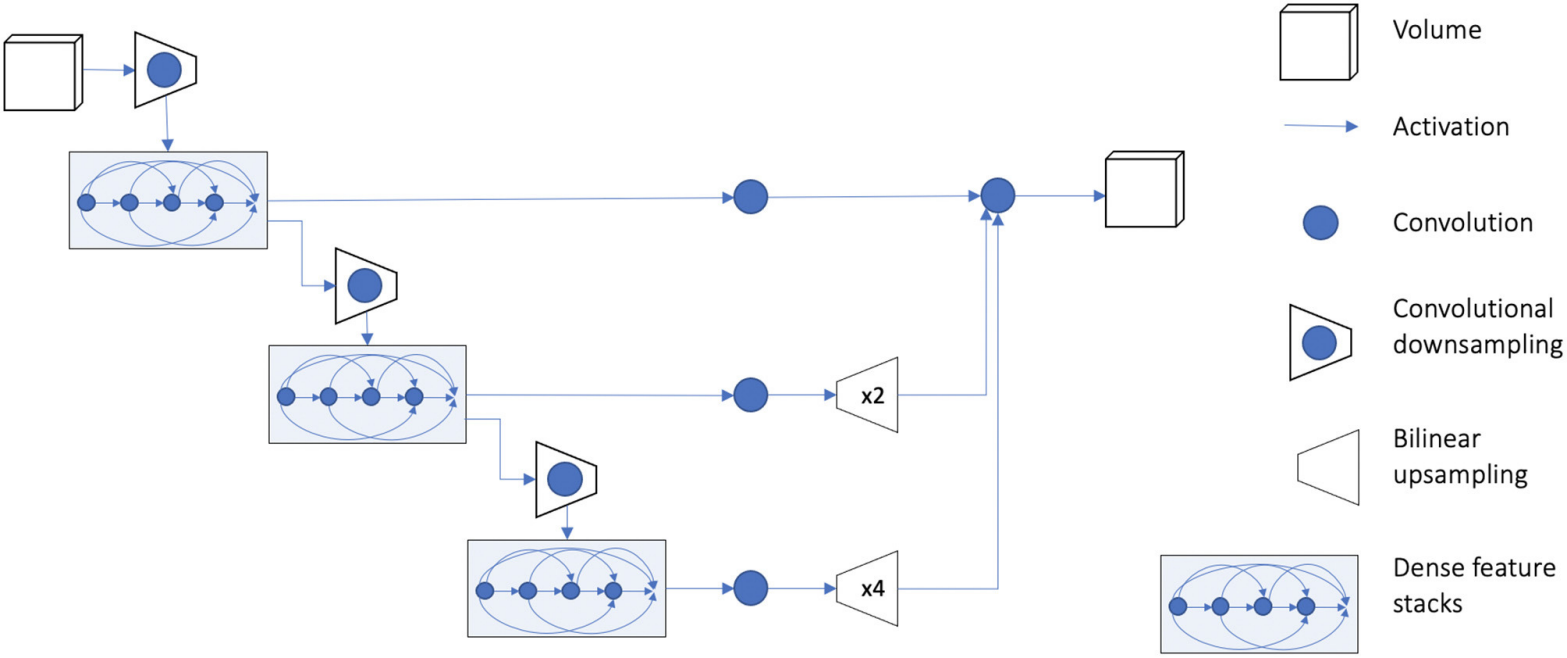
Overview of model architecture. Detailed description of the model architecture is available in Gibson et al. (2018a). The output of the model is resized to the original input image dimension during postprocessing. An implementation of the model is available in the NiftyNet platform (http://niftynet.io) in code repositories.

We used TensorFlow (version 1.12.0) and the medical imaging deep learning platform NiftyNet (Li et al., 2017; Gibson et al., 2018b; version 0.6.0) for implementation of all experiments. NiftyNet is a modularly structured deep learning platform tailored toward medical image analysis applications with modules for preprocessing, network training, evaluation, and inference. Minimal coding is required from the user using this platform, and the specific settings related to preprocessing images, training, and testing can be communicated via a configuration file. We used the 3D fully CNN (Long et al., 2015) architecture known as dense V-network (Dense-Vnet) that has previously demonstrated success in establishing voxel-to-voxel connections between input and output images in multiorgan segmentation of abdominal computed tomography images (Gibson et al., 2018a). The architecture of the model is shown in Figure 3, and it only differs from the original model in the size of input image (in our case, 240 × 240 × 64) and the lack of priors. The encoder block of the segmentation network generates three different sized sets of feature maps using dense feature stacks (Huang et al., 2017). The outputs are upsampled using the decoder block so that the smaller feature maps match the original input size. The final output is the concatenated version of all outputs after a single convolution in the skip connection. It should be noted that the Dense-Vnet architecture is designed to work with a smaller version of the original image to constrain memory usage (i.e., the first convolutional downsampling layer in Figure 3), and the final output is resized to the original image size during postprocessing. An implementation of the model and post-processing is available in the NiftyNet platform (http://niftynet.io).

Hyperparameters included learning rate, optimizer, and augmentation, which were selected using the validation set. Training was conducted using He weight initialization (He et al., 2015), whitening (scaling image intensities to 0–1), adam (Kingma and Ba, 2014) optimizer with a batch size of 6, and the Dice coefficient as the loss criteria (Milletari et al., 2016). We trained the model for a maximum of 300 iterations, and the model that performed best on the validation set was used as the final model. It should be added that the results reported here were generated without the use of any augmentation as data augmentation (including rotation, scaling, and flipping images on the *x*-axis) did not improve model performance on the validation set. All experiments were conducted on an Ubuntu 17.10 system with a single Nvidia TITAN V GPU. The source code for NiftyNet platform along with instructions on how to call the platform via terminal is available at: https://github.com/NifTK/NiftyNet.

Our trained models along with the complete list of parameters utilized for model training are available at: https://github.com/SARARANJBAR/skullstripping_niftynet.

### Experiments

Using only brain tumor data, we evaluated the performance of the network across MRI contrasts by repeating model training three times: first using only T1Gd MRIs, second using only FLAIR MRIs, and finally using both series as inputs. When both T1Gd and FLAIR sequences were provided to the network as input, the two images were simultaneously provided to the model. Apart from input image type, all other training parameters were identical between different runs. We evaluated model performance using Dice similarity coefficient (Kingma and Ba, 2014), sensitivity, specificity, and Hausdorff distance (Kingma and Ba, 2014), comparing predicted labels with manual brain masks. Sensitivity measures the detection rate of brain tissue, and specificity measures how much non-brain tissue is correctly identified, whereas Dice score evaluates the trade-off between sensitivity and specificity, measuring the overlap of predictions and ground truths. Hausdorff distance measures the Euclidean distance between the farthest contours of the ground truth and predictions and is relevant to this work to assess accuracy of predictions at the edge of the brain.

In addition to brain tumor data, we used the healthy subject data from LBPA40 (Gibson et al., 2018b) dataset to evaluate the performance of trained models on a publicly available benchmark. Other deep-learning skull stripping methods in the literature (Chang et al., 2009; Kleesiek et al., 2016; Mohseni Salehi et al., 2017; Lucena et al., 2019) have used this data collection to evaluate their model. Although our model was not trained on healthy subjects, we believe addition of this experiment will help place our work within existing literature. Average Dice score was used as the performance measure. The Dice scores of previous approaches were acquired from their publications.

## Results

**Figure 4:**
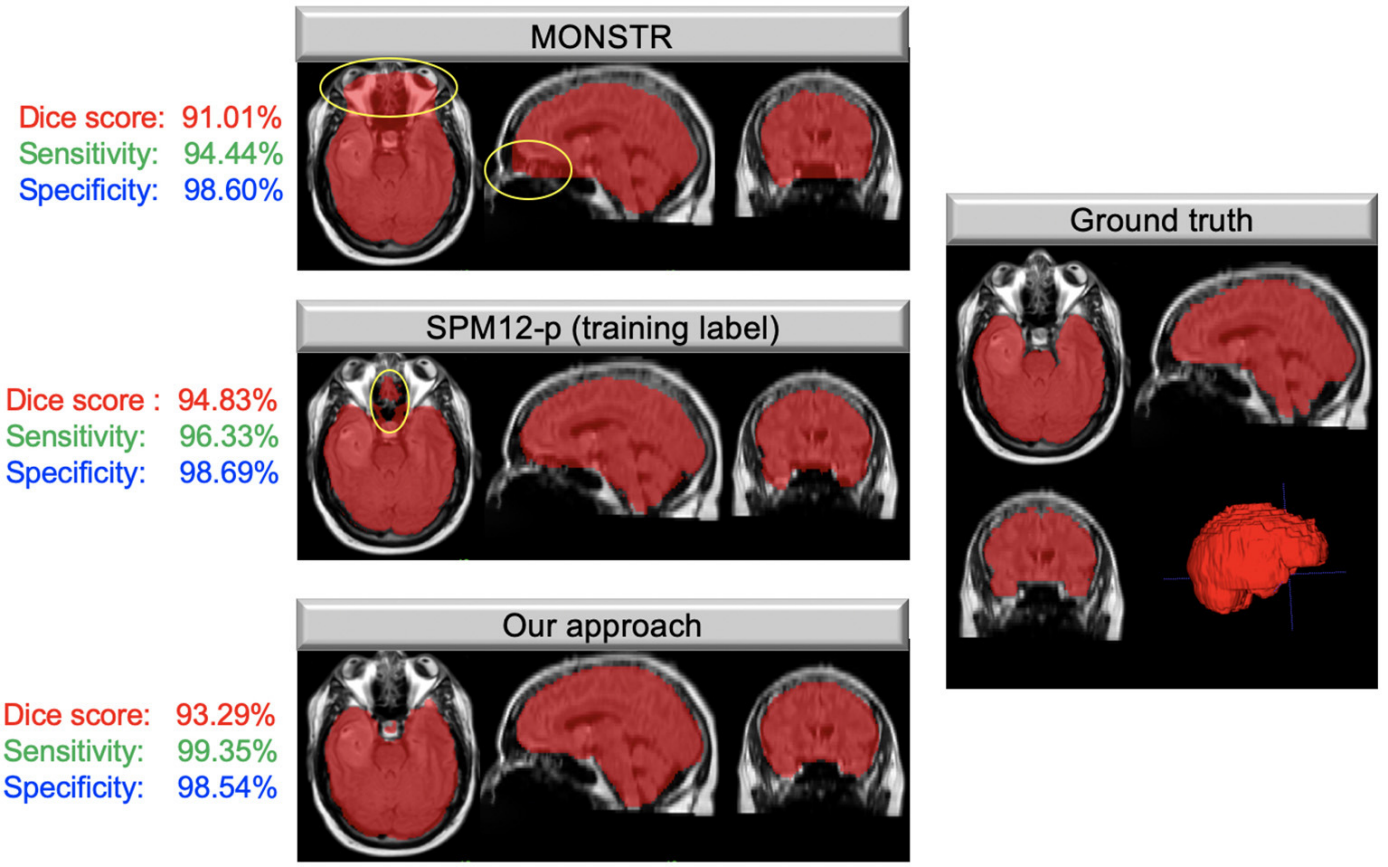
Masks overlaid on brain tumor MRIs; images on the left show the brain masks created using MONSTR, SPM12-p, and our model in different anatomical views. Areas highlighted in yellow show errors in results. The right image shows the ground truth manual segmentation. Our approach performed very well and much better than the other two methods. The Dice coefficient, sensitivity, and specificity, calculated based on the ground truth for this case, are shown to the left of each image.

Table 1 compares the performance of model training on brain tumor data across input types on previously unseen test cases with available ground truth. We found the model trained on FLAIR to achieve the highest Dice score and sensitivity, and the model trained on both sequences was superior to single input models in specificity (98.84%). Our FLAIR-only model achieved a mean Dice score of 94.54%, a sensitivity of 96.39%, and specificity of 98.48% on the test set with available ground truth. The average Dice score for the FLAIR-only model was not significantly higher than that of the model trained on both sequences (*p* = 0.83, *t*-test) but was significantly higher than that of the T1Gd-only model (*p* = 0.00042), which was also significantly outperformed by the model trained on both (*p* = 0.0027). The model trained on both modalities achieved a slightly higher but non-significant mean specificity than the FLAIR-only model (*p* = 0.14), with the FLAIR model significantly outperforming the model trained on both in mean sensitivity (*p* = 0.043). The T1Gd model was significantly lower in mean specificity than the model trained on both modalities (*p* = 0.0016) and lower than the model trained only on FLAIR; this result was not significant (*p* = 0.068). The T1Gd-only model had a slightly lower mean sensitivity than the FLAIR-only model (*p* = 0.7612). The average Hausdorff distance between the predictions of the FLAIR model and ground truth was also superior to that of T1Gd-only (*p* = 0.023) and dual input (T1Gd + FLAIR) models (*p* = 0.71). Table 2 compares the performance of our model with non-learning methods MONSTR and SPM12. While MONSTR did not fail to include the regions occupied by tumors into the segmentation, its performance was much worse in identifying the boundaries of the brain in other regions, and oversegmentation and undersegmentation were observed at the top and bottom slices. In comparison, SPM12-p showed a much improved sensitivity. Our model was superior in Dice score, Hausdorff distance, and sensitivity compared with both non-learning approaches. An example of predicted brain mask and comparison with MONSTR and SPM12 is presented in Figure 4. Using the same machine for training, generating an SPM12-p mask required an average of 2–3 min compared with 10–20 min for MONSTR, and 2–3 s for the model. Longer runtime is expected for MONSTR as atlas-based methods tend to take longer than other approaches.

**Table 1:** Comparison of model performance across input type on the test set.

**Model input**	**Dice score**	**Sensitivity**	**Specificity**	**Hausdorff distance**
T1Gd	93.09 (1.78)	96.14 (3.81)	97.92 (1.28)	3.69 (0.55)
FLAIR	**94.54** (1.09)	**96.39** (2.34)	98.48 (1.05)	**3.39 (0.44)**
T1Gd + FLAIR	94.47 (1.61)	94.80 (3.49)	**98.84** (0.79)	3.44 (0.49)

*Values indicate mean and standard deviation. Best result is highlighted in bold font*.

**Table 2:** Comparison of performance between model and non-learning methods on the test set.

**Method**	**Dice score**	**Sensitivity**	**Specificity**	**Hausdorff distance**
MONSTR	91.34 (6.76)	88.22 (7.44)	**98.91 (2.22)**	3.67 (0.75)
SPM12-p	93.36 (3.75)	93.39 (6.59)	98.76 (1.05)	3.44 (0.80)
Our approach	**94.54 (1.09)**	**96.39 (2.34)**	98.48 (1.05)	**3.39 (0.44)**

*Values indicate mean and standard deviation. Best result is highlighted in bold font*.

**Figure 5:**
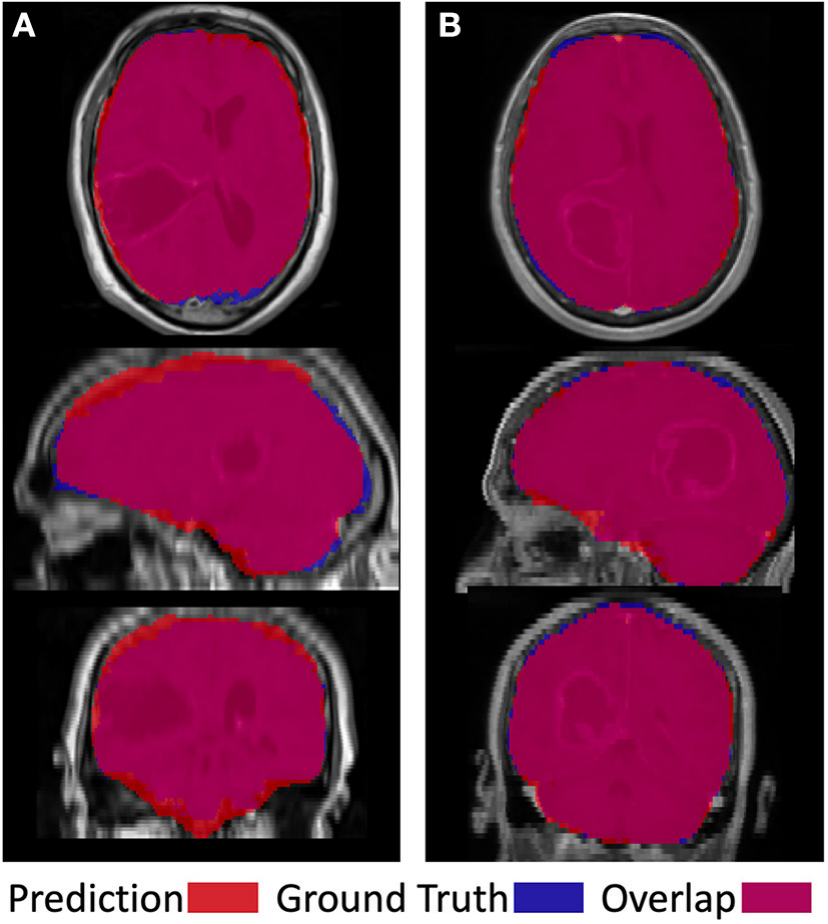
Visual examples of a less successful case **(A)** and a more successful case **(B)**. Prediction is shown in red, ground truth in blue, and overlap in purple.

Figure 5 shows two examples of a model prediction (red), ground truth (blue), and overlap (purple) (left). This prediction achieved a relatively low Dice score of 92.4%, with areas of both underprediction and overprediction. In this case, the model more commonly underpredicted the anterior and posterior regions of the brain, while overpredicting the superior and inferior regions. This prediction achieved a relatively high Dice score of 96.6%, primarily underpredicting the superior region and overpredicting the inferior regions. Importantly, there is no evidence that the net suffered from the presence of tumor abnormalities in either case.

Table 3 presents the performance of our model on healthy subjects. On average, our model achieved a Dice score of 96.2%, sensitivity of 96.6%, and specificity of 99.2% on the LBPA40 dataset. Overall, our results were within the range of those reported by others in similar applications. However, our Dice score and sensitivity were on the lower end of scores. We believe this is expected given that, unlike others, we trained our model using brain-tumor patient data that divert from the normal brain due to imaging patterns resulting from pathology.

**Table 3:** Comparison of performance with previous literature on healthy brains from the LBPA40 dataset.

**Method**	**Dice score**	**Sensitivity**	**Specificity**
CONSNet (Milletari et al., 2016)	97.35 (0.003)	97.26 (0.007)	**99.54 (0.001)**
Auto-U-Net (Mohseni Salehi et al., 2017)	**97.73 (0.003)**	**98.31 (0.006)**	99.48 (0.001)
U-Net (Mohseni Salehi et al., 2017)	96.79 (0.004)	97.22 (0.016)	99.34 (0.002)
3D CNN (Kleesiek et al., 2016)	96.96 (0.010)	97.46 (0.010)	99.41 (0.003)
Our approach	96.17 (0.220)	96.60 (0.080)	99.22 (0.090)

*Performance measures of others' works are extracted from their publication. Values in bold font indicate the best result*.

## Discussion

Despite the large body of existing literature on automatic skull striping methods on MRI, few have reported robustness in the presence of a pathology (Thakur et al., 2019). The closest work to ours is the modality-agnostic 3D CNN created by Thakur et al., Lucena et al. (2019), which was tested on brain tumor data from three different institutions compared with ours with 20+ institutions. Authors trained their network with pretreatment images of glioma patients using T1-weighted, T1Gd, T2-weighted, and FLAIR sequences. Their model achieved an average Dice coefficient of 97.8% on images from the training institution and 95.6, 91.6, and 96.9% on datasets of other institutions. Another learning-based skull stripping approach is the work of Kleesiek et al. (2016), in which authors created a modality-agnostic fully convolutional CNN model with similar input channels as Thakur et al. and achieved an average Dice of 95.2% and a sensitivity of 96.25% on a cohort of 53 brain tumor patients from training institution. Our work differs from these works (Kleesiek et al., 2016; Thakur et al., 2019) in a number of ways. First, our approach is considered weakly supervised, as the network was trained using automatically generated labels with known imperfections (Malone et al., 2015) compared with accurate ground truth delineated by neuroradiologists. The data used in this work were collected at 20+ institutions from 1990 to 2016 using a variety of imaging devices that has been shown to impact the outcome of skull stripping (Rex et al., 2004; Fennema-Notestine et al., 2006). However, we argue that an advantage of this type of data heterogeneity is that it better approximates the data found in clinical practice and therefore can serve as a realistic benchmark for estimating model performance in clinical practice. The fact that our result is within the range of reported performance in Thakur et al. (Lucena et al., 2019) on data from other institutions is a good indicator for this argument. Given that the CSF is dark on both FLAIR and T1Gd images, and brain tissue is brighter than CSF on both images, the major visual difference between the two images is the high intensity of skull on T1Gd and its low intensity the FLAIR image. This can result in a sharper edge at the boundary of the brain on the FLAIR images, which we associate with the improved performance of the FLAIR model. That said, given the small size of our test set and similarly promising results of our other models, we urge the reader not to discount models trained only on T1Gd or a combination of images. One limitation of our work is that we did not train a sequence-agnostic model. In our results, the FLAIR model yielded the highest Dice and sensitivity, and the addition of T1Gd slightly improved specificity. Given the heterogeneity of data types across institutions, a sequence-agnostic approach is beneficial for ensuring utility across data found in clinical practice, and we intend to adopt a similar approach in future work.

Because of the size of our cohort and the labor-intensive nature of manual segmentation, we needed an automatic method to create brain masks for training. We selected SPM12 because of its reported comparable performance with manual delineation in segmenting total intracranial volume on MRI even in the presence of neurodegenerative pathology (Malone et al., 2015). Compared with ground truth, the SPM12-p labels achieved a Dice of 93.34% on the test set. Visualization of model output against ground truth showed the net was not hindered by the presence of tumor abnormalities; rather, the differences in Dice score were related to the overall brain shape. Despite the reported high performance of MONSTR in skull stripping brain tumor data, we found its performance worse than SPM12, demonstrated by comparing the Dice score of generated masks with ground truth (Table 2). As a result of this finding, we decided to proceed with model training with SPM12. However, no single automatic method for generating labels can outperform consensus methods that combine different skull-stripping methods through a meta-algorithm and allow for combining the strength of different approaches. In the work of Lucena et al. (Milletari et al., 2016), the authors generated silver standard labels for training using the STAPLE (Warfield et al., 2004) method combining eight different segmentation approaches into a probabilistic consensus mask, and achieved a Dice score of 97.3% and sensitivity of 97.2% on healthy subjects. In comparison, our approach could be considered a “bronze standard” given that our labels were acquired using one segmentation method. In future work, we aim to repeat our analysis using a silver standard.

Among the non–learning-based skull stripping approaches in the literature, the MONSTR algorithm (Roy et al., 2017) was reported to outperform other methods on a small cohort of five brain tumor cases with an average Dice agreement of 96.95% with ground truth. MONSTR achieved a moderate Dice score of 91.34% on the test set. In comparison, SPM12-p outperformed MONSTR, particularly with respect to sensitivity (93.39 vs. 88.22%), as well as average runtime for creating masks (2–3 vs. 10–20 min on the machine used for model training). Discrepancy between the results here and the reported performance in the original paper could also be related to our use of T1Gd and FLAIR inputs for creating MONSTR masks, as opposed to T1Gd and T2W images that were used in the original results (Roy et al., 2017). The worse performance by MONSTR could also be associated with the atlas-based nature of the algorithm, which can result in inaccuracies when images deviate from healthy brain MRIs. The performance of our model on healthy subjects was decidedly on the lower end of reported results for deep learning–based skull stripping models in the literature. Mohseni Salehi et al. (2017) compared the performance of a voxel-wise approach using three convolutional pathways for each anatomical plane and a fully convolutional U-Net (Ronneberger et al., 2015) architecture and achieved Dice coefficients of 97.7 and 96.8% on two publicly available datasets of normal brains. Although the authors used the U-Net architecture, which might be considered dated in today's deep learning context, their approach achieved a higher performance than ours because of their use of different convolutional pathways for each anatomical plane. Kleesiek et al. (2016) used a 3D input-agnostic fully convolutional network and compared its performance to six other skull stripping methods on publicly available datasets. Whereas, Kleesiek et al. (2016) reported the performance of their model on merged public datasets, others (Lucena et al., 2019) reported their performance on the LBPA40 dataset alone to be an average Dice score of 97.0% and sensitivity of 97.4%. Lucena et al. (Milletari et al., 2016) adopted a brain extraction model consisting of three parallel, fully convolutional networks using the U-Net architecture and achieved a Dice score of 97.3% and sensitivity of 97.2%. Here again, the authors utilized parallel pathways to achieve high performance. Our approach did not yield the same level of Dice score on the LBPA40 dataset. We believe this is expected given that unlike others we trained our network using only brain-tumor MRI and did not use manually delineate or consensus methods for training labels. In future work, we intend to adopt a consensus method for creating training labels. To maximize generalizability and utility of this tool, we will supplement brain tumor data with healthy subjects to improve model performance on healthy subjects as well as to stay relevant for utility in clinical settings. In addition to using pathological MRI for model training with suboptimal labels, we adopted a straightforward volumetric training approach with no pathway parallelization for different anatomical planes. This could also explain the drop in our model performance compared with others.

In summary, we assessed the performance of a deep learning model in MRI brain extraction of a diverse multi-institutional brain tumor patient dataset using weak labels. On previously unseen brain tumor cases, our approach reached comparable performance to previous literature. The model underperformed compared with state-of-the-art models in the literature on healthy subjects, which can be attributed to the absence of healthy patients in our training set and our rather simplistic model training approach. The shortcomings can be addressed by fine tuning the model on healthy subjects, leveraging a consensus approach to generating training labels, and allocating training pathways within the model for different anatomical planes. Despite the shortcomings, we believe that our approach can be a practical choice for skull stripping MRI data in repositories of brain tumor patients given its turnaround time and simplicity. In future work, we intend to extend this work to perform skull striping on post-treatment MRIs.

## Data Availability Statement

The data analyzed in this study is subject to the following licenses/restrictions: MR imaging data of brain tumor patients used in this study was acquired from our in-house IRB-approved repository which contains patient information and therefore is subject to HIPAA regulations. Due to the proprietary nature of patient data and patient information, we are not at liberty to freely share data with readers. However, data may be available for sharing upon the request of qualified parties if patient privacy and intellectual property interests of our institution are not compromised. Typically, data access will occur through collaboration and may require interested parties to obtain an affiliate appointment with our institution prior to data access. Requests to access these datasets should be directed to https://mathematicalneurooncology.org. Healthy subject data used in this work were acquired from the publicly available LBP40A dataset. Transforms from delineation and native radiological spaces are available on The LONI Probabilistic Brain Atlas Project webpage at: https://resource.loni.usc.edu/resources/atlases-downloads/.

## Ethics Statement

All procedures performed in the studies involving human participants were in accordance with the ethical standards of the institutional and/or national research committee and with the 1964 Helsinki declaration and its later amendments or comparable ethical standards. Our de-identified data repository of patients with brain cancer includes retrospective data collected from medical records and prospective data collection. Research on the data repository was reviewed and approved by Mayo Clinic Institutional Review Board. Prior to collection of retrospective data, informed consent was waived for those participants by the Mayo Clinic Institutional Review Board (IRB# 15-002337). Written informed consent was obtained for all prospectively enrolled participants as approved by Mayo Clinic Institutional Review Board (IRB# 17-009682). The patients/participants provided their written informed consent to participate in this study.

## Author Contributions

SR, KWS, JRM, and KRS contributed to study design. SR led model generation and data processing as well as writing the first draft of the manuscript. KWS and KRS created the infrastructure necessary for conducting the study. SR, KWS, JRM, LC, CRR, and LEP contributed to data collection and data preprocessing. LSH was the clinical lead of the study and reviewed the accuracy of the ground truth brain masks. JRM and KRS share senior authorship. All authors have substantially contributed to conducting this research and drafting the manuscript. All authors have edited the manuscript and have approved the contents.

## Funding

This publication would not have been possible without the support of the James S. McDonnell Foundation, the Ivy Foundation, the Mayo Clinic, the Zicarelli Foundation, and the NIH (R01 NS060752, R01 CA164371, U54 CA210180, U54 CA143970, U54 CA193489, U01 CA220378, and U01 CA250481-02).

## Conflict of Interest

The authors declare that the research was conducted in the absence of any commercial or financial relationships that could be construed as a potential conflict of interest.

## Publisher's Note

All claims expressed in this article are solely those of the authors and do not necessarily represent those of their affiliated organizations, or those of the publisher, the editors and the reviewers. Any product that may be evaluated in this article, or claim that may be made by its manufacturer, is not guaranteed or endorsed by the publisher.
